# Apolipoprotein L3 inhibits breast cancer proliferation and modulates cell cycle via the P53 pathway

**DOI:** 10.7150/jca.96903

**Published:** 2024-07-02

**Authors:** Hao Yu, Siyan Li, Xing Li, Yanbiao Liu, Zhaobu Wang, Mengyao Cui, Feng Jin, Xinmiao Yu

**Affiliations:** Department of Breast Surgery, The 1st Affiliated Hospital of China Medical University, Shenyang, Liaoning, China.

**Keywords:** Apolipoprotein, Proliferation, Cell cycle, Y-box binding protein 1, P53, Breast cancer

## Abstract

**Background:** Breast cancer is the second most common cause of cancer-related mortality globally. Apolipoprotein L3 (APOL3), a member of the apolipoprotein family, has been implicated in the pathogenesis of cardiovascular diseases. Nevertheless, the functions and underlying mechanisms of APOL3 in breast cancer have yet to be elucidated.

**Methods:** The patient data were sourced from The Cancer Genome Atlas (TCGA) and Gene Expression Omnibus (GEO) database. Quantitative real-time PCR (qRT-PCR), western blotting, and immunohistochemistry (IHC) assays were used to assess expression of APOL3. Cell proliferation rates were determined by Cell Counting Kit-8 (CCK-8) and colony formation assays. Flow cytometry was used to examine cell cycle distribution. Western blotting was conducted to investigate the expression of cell cycle related proteins. A xenograft model was used to evaluate the effect of APOL3 *in vivo*. APOL3-binding proteins were identified through mass spectrometry, co-immunoprecipitation (CO-IP) assay and immunofluorescence assay.

**Results:** APOL3 expression was significantly downregulated in breast cancer, and its low expression was correlated with poor prognostic outcomes. Overexpression of APOL3 suppressed breast cancer cell proliferation, induced cell cycle disruption. Conversely, knockdown of APOL3 promoted cell proliferation. *In vivo* animal experiments demonstrated that APOL3 overexpression can inhibit tumor proliferation. Mass spectrometry, CO-IP and immunofluorescence assay confirmed the interaction between APOL3 and Y-box binding protein 1 (YBX1). Furthermore, YBX1 knockdown following APOL3 knockdown mitigated the enhanced proliferation. These results provide new ideas for clinically targeting APOL3 to inhibit proliferation in breast cancer.

**Conclusions:** Our findings indicate that APOL3 inhibits breast cancer cell proliferation and cell cycle modulating P53 pathway through the interaction of YBX1.

## Introduction

Breast cancer has now surpassed lung cancer as the most prevalent malignancy globally 1. The American Cancer Society reported that in 2022, there were 287,850 new breast cancer cases (constituting 31% of all female cancers) and 43,250 fatalities attributed to the disease worldwide 2. In China, the incidence of breast cancer is rising at a rate approximately double that of the global average each year 3. Despite advances in early detection and the improved efficacy of comprehensive treatments, breast cancer patients continue to confront high rates of recurrence and concerning prognoses 4, 5. In recent years, with the development of biotechnology, new biomarkers have brought new hope for the diagnosis and treatment of breast cancer. As the sustained activation of proliferative signals during the occurrence and development of cancer is a recognized cancer hallmark 6, 7, finding initiation factors driving tumor proliferation is crucial for developing precise strategies.

Apolipoprotein L3 (APOL3) belongs to the apolipoprotein L gene family and is located at chromosome 22q12.3. This family plays a role in the transport and metabolism of lipids 8. However, recent studies have found that they have some additional functions besides their involvement in lipid metabolism. Among their family members, APOL1 and APOL3 are both proteins that can induce cell death and are associated with the endoplasmic reticulum and Golgi apparatus membranes, capable of forming potassium channels. Uniquely, APOL3 can regulate the neuronal calcium sensor-1 (NCS-1), facilitate the interaction between NCS-1 and PI4KB, enhance PI4KB activity, and consequently influence the migration and survival of cancer cells 9. APOL proteins were found to be implicated in a variety of solid tumors. For example, APOL1 is related to cell homeostasis, and its overexpression could induce autophagy and autophagy-associated cell death to maintain the homeostasis of renal cells, thereby inhibiting the occurrence of renal cell carcinoma 10. Interestingly, APOL1 is overexpressed in thyroid cancer and seems to be involved in tumor progression through repression of the regular apoptotic program 11. In bladder cancer, APOL2, as a member of the risk staging model, could predict the prognosis of bladder cancer 12. In prostate cancer, APOL3 locus was found to span two linkage disequilibrium blocks and is considered a risk locus 13. Both APOL3 and APOL6 have been reported in colorectal cancers 14, 15. Among them, APOL3 promotes ferroptosis of colorectal cancer cells and antitumor ability of CD8+ T cells and is an intervention target of CRC 15. APOL6 is a prognostic gene associated with ferroptosis in colon cancer. Knockout of APOL6 reduces apoptosis 16. Moreover, APOL3 was recently featured in a study that developed a predictive gene panel for breast cancer, which included a total of ten lipid-metabolism-related genes and demonstrated a robust capacity to predict the overall survival of patients 17. Despite these developments, the biological functions and molecular mechanisms of APOL3 in breast cancer remain to be elucidated.

This study aimed to investigate the biological function of APOL3 in breast cancer and to unravel its regulatory mechanisms in detail. We conducted *in vitro* experiments to assess the effect of APOL3 on the proliferation and cell cycle dynamics of breast cancer cells. Through co-immunoprecipitation (CO-IP) assays and mass spectrometry, we identified an interaction between APOL3 and Y‑box binding protein 1 (YBX1) and further investigated the downstream pathways involved. Our study contributes novel insights into the tumor-suppressive function of APOL3 in breast cancer.

## Methods

### Dataset acquisition and bioinformatics analysis

We accessed the data of 1083 breast cancer patients from The Cancer Genome Atlas (TCGA) (https://tcga-data.nci.nih.gov/), presented as fragments per kilobase of exon per million mapped fragments (FPKM). We also downloaded the GSE29431 series (https://www.ncbi.nlm.nih.gov/geo/query/acc.cgi?acc=GSE29431) and GSE42568 series (https://www.ncbi.nlm.nih.gov/geo/query/acc.cgi?acc=GSE42568) from the Gene Expression Omnibus (GEO) database. For survival analysis and the construction of receiver operating characteristic (ROC) curves, we utilized the “Survminer”, “Survival”, “pROC”, and “ggplot2” packages within R (3.6.3) software.

### Cell lines and culture

The human normal mammary epithelial cell line MCF10A and the breast cancer cell lines MCF7, T47D, MDA-MB-231, MDA-MB-468, and BT-549 were procured from the American Type Culture Collection (ATCC). The MCF10A cells were maintained in a specific medium formulated for this cell line, acquired from Procell. The MCF7 cells were cultured in high glucose Dulbecco's Modified Eagle's Medium (DMEM) containing 10% fetal bovine serum (FBS) and 1% penicillin/streptomycin. The MDA-MB-231 and MDA-MB-468 cells were cultured in Leibovitz's L15 medium containing 10% FBS. Both T47D and BT-549 cells were cultured in RPMI-1640 medium containing 10% FBS and 1% penicillin/streptomycin. The DMEM and RPMI-1640 media were sourced from Hyclone, and Leibovitz's L15 medium was supplied by KeyGEN BioTECH.

### RNA extraction and Quantitative Real-time PCR

Total RNA was extracted from cell samples using TRIzol reagent (Invitrogen, USA), and mRNA was subsequently reverse-transcribed into cDNA using ReverTra Ace™ qPCR-RT Master Mix (Toyobo, Japan), as per the manufacturer's instructions. Quantitative real-time PCR (qRT-PCR) assays were conducted using SYBR qPCR Mix (Toyobo, Japan) in a 13μL reaction volume, with the amplification process spanning 40 cycles. The relative expression levels of the target mRNAs were determined using the 2^-∆∆Ct^ method. Primer sequences used in this study are detailed in Table 1.

### Immunohistochemistry

All of the tissues were obtained with informed consent from all patients and their families, and approved by the First Affiliated Hospital of China Medical University ethics committee (approval: AF-SOP-07-1.1-01). Breast cancer and normal mammary tissues were sectioned at 4mm thickness and baked at 65 °C for 4h. The sections underwent deparaffinization in xylene for 10 minutes, repeated twice, followed by antigen retrieval in citrate buffer (pH = 6) or Tris-EDTA (pH=9) for 10 minutes. The primary antibodies used were APOL3 (ab154869, Abcam,1:200), Ki-67 (27309-1-AP, Proteintech, 1:5000), CDK6 (66278-1-Ig, Proteintech,1:200), Cyclin A2 (66391-1-Ig, Proteintech, 1:2000) and Cyclin D1 (26939-1-AP, Proteintech, 1:1500), which were incubated with the tissue sections at 4 °C overnight. Subsequent color development was achieved using DAB and hematoxylin, and the sections were dehydrated after acidification with hydrochloric acid ethanol. Both the DAB and immunohistochemical (IHC) kit were procured from Fuzhou Maixin Biotech (China).

### Protein extraction and Western blotting

Protein extraction was performed using radioimmunoprecipitation assay (RIPA) buffer (Beyotime, China) supplemented with a protease inhibitor cocktail (Sigma, USA). Protein concentrations were determined using the BCA Protein Assay Kit (Beyotime, China) following the provided instructions. The proteins were separated by SDS-PAGE and subsequently transferred to polyvinylidene fluoride (PVDF) membranes (Millipore, German). The primary antibodies used were APOL3 (ab154869, Abcam), GAPDH (ab181602, Abcam), β-actin (66009-1-Ig, Proteintech), CDK6 (66278-1-Ig, Proteintech), Cyclin A2 (66391-1-Ig, Proteintech), Cyclin D1 (60186-1-Ig, Proteintech), P53 (ab26, Abcam) and P21 (ab109520, Abcam). After incubation with appropriate secondary antibodies, protein detection was achieved using enhanced chemiluminescence (ECL) with BeyoECL Plus (Beyotime, China).

### Plasmid engineering and cell transfection

Lipofectamine 3000 (Invitrogen, ThermoFisher Scientific) was used for the transient transfection of MCF7 and MDA-MB-231 cell lines. The APOL3 overexpression plasmid (OE-APOL3) and the corresponding negative control (NC) plasmid were engineered by GenePharma (China). The sequences of the APOL3-targeting siRNA were 5'‑ GAC CGA UUG AAG GUA UUU ATT‑3' and 5'‑ CAG CUA UUG AGG ACG AAU ATT‑3'. The sequence of the YBX1-targeting siRNA was 5'‑CAG UUC AAG GCA GUA AAU AUG CA‑3'. At 48h and 72h post-transfection, RNAs and proteins were harvested for the assessment of transfection efficiency using qRT-PCR and western blotting.

### CCK-8 assay

The Cell Counting Kit-8 (CCK-8) assay was used to assess cell proliferation. MCF7 and MDA-MB-231 cells transfected for 48h were seeded uniformly into 96-well plates and maintained at 37 °C. CCK-8 solution (APExBIO, America) was added at 0h, 24h, 48h, and 72h post-seeding, followed by a 2h incubation period. Subsequently, the absorbance was measured at a wavelength of 450nm to determine cell proliferation rates.

### Colony formation assay

Following transfection with OE-APOL3 and NC, cells were trypsinized and counted 48h later. A total of 1×10^3 cells were seeded into 35-mm Petri dishes and cultured at 37 °C for 2 weeks. After incubation, the culture medium was discarded, and the colonies were fixed with 4% polyformaldehyde. After washing three times with PBS, colonies were stained with 0.1% crystal violet. Colonies consisting of more than 50 cells were counted.

### Cell cycle analysis

After transfection for 24h, both MCF7 and MDA-MB-231 cells were harvested and treated with RNase A (Bestbio, China) at 37 °C for 30 min. Subsequently, propidium iodide (Bestbio, China) staining was performed, followed by a 5-min incubation at 4 °C for 5 minutes. Fluorescence intensities were then measured to analyze the cell cycle distribution.

### CO-IP assay

Transfected MCF7 cells were collected and lysed using the buffer (Beyotime, China) supplemented with PMSF (Beyotime, China), with the lysates incubated at 4 °C for 4h. The lysates were aliquoted into three tubes, one serving as the input (positive control). MYC antibodies (16286-1-AP, Proteintech) or YBX1 antibodies (20339-1-AP, Proteintech) were added to the experimental tube, while IgG served as the negative control. All were incubated with rotation overnight at 4 °C. Agarose beads (Thermo Fisher Scientific, USA) were then added and incubated with rotation at 4 °C for an additional 3h. PBS was used to wash the agarose beads. The protein complexes bound to the beads were analyzed via western blotting to investigate protein interactions.

### Immunofluorescence assay

Cells were fixed by 4% paraformaldehyde and permeabilized using 0.3% Triton-PBS (Beyotime, China). 5% BSA was used to block cells and anti-APOL3(YT0279, Immunoway) and anti-YBX1 (sc-101198, Santa Cruz) were added for overnight incubation in 4 °C. And cells were incubated with secondary antibodies for 2h at room temperature. DAPI was used to observe the nucleus and slides were observed under confocal microscope.

### *In vivo* animal experiments

A total of 10 five-week-old female Balb/c-nude mice, weighing 17-18 g each, were used for this study, with five mice in each group. These mice were obtained from Beijing HFK Bioscience Co., Ltd. and housed under specific pathogen-free (SPF) conditions with access to standard diet and water. The experimental cohorts were established as the NC group and the OE-APOL3 group. The study protocol was approved by the Animal Research Ethics Committee of China Medical University (approval: CMU20231000). Each mouse was subcutaneously inoculated with 5×10^6 cells from either the NC or OE-APOL3 modified MCF7 cell lines. Body weight and tumor growth were measured every 2 days and tumor volume was calculated using the formula: 0.5×Width^2^×Length. After the experiment, all mice were humanely euthanized using carbon dioxide. Tumor specimens were then meticulously harvested, fixed in formalin at room temperature, and subsequently embedded in paraffin for future histological analysis.

### Statistical analysis

The data were presented as the 'mean ± standard deviation (SD)'. Each assay was independently replicated at least three times. The Student's t-test was employed for comparisons between two groups, while one-way ANOVA was used for comparisons among multiple groups. All statistical analyses were conducted using GraphPad Prism 7.0 software. A *p*-value less than 0.05 was deemed to indicate statistical significance.

## Results

### APOL3 expression is diminished in breast cancer tissues compared with normal mammary tissues

**Figure 1:**
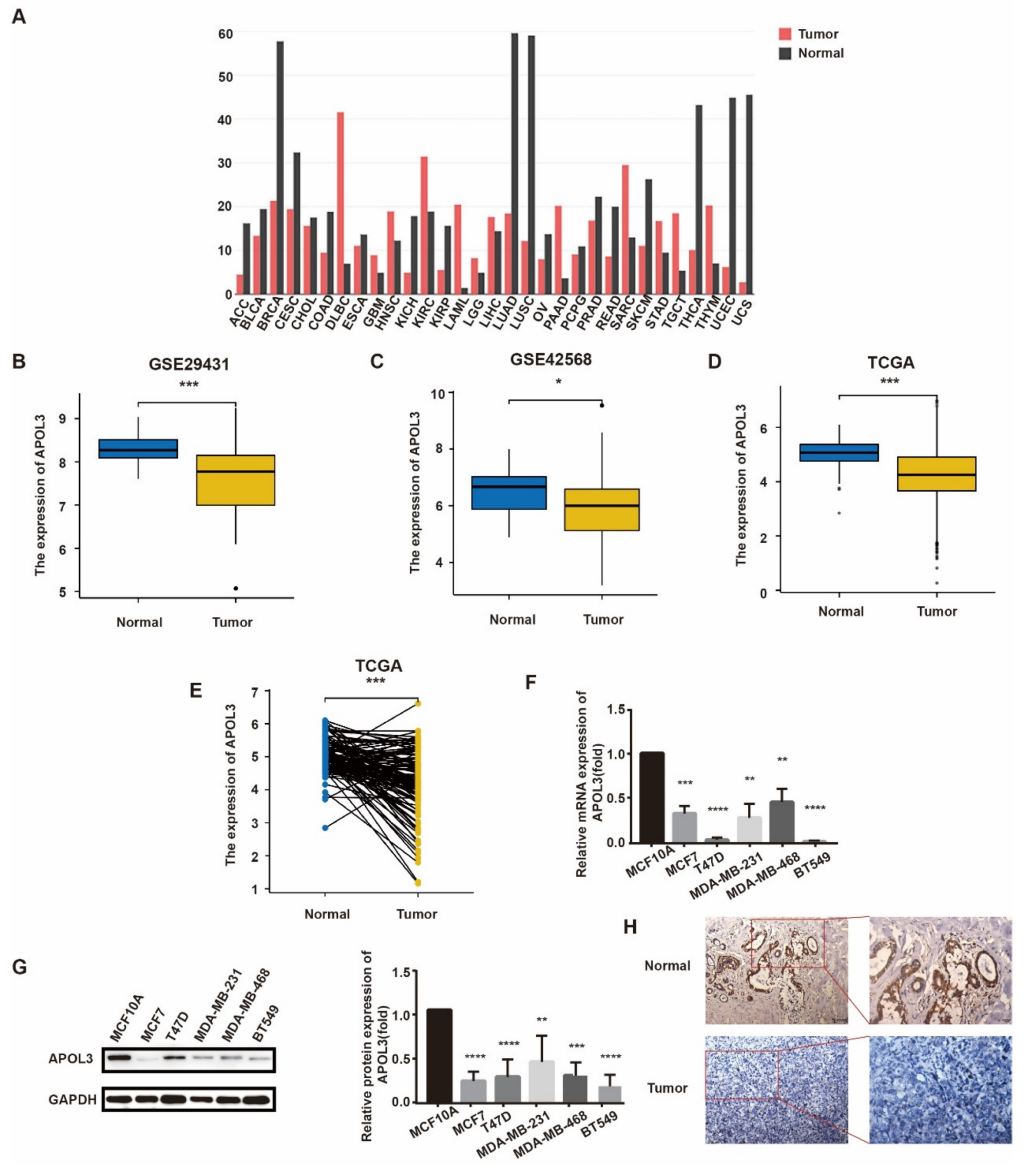
APOL3 Expression in normal and breast cancer tissue. A: Pan-cancer expression profile of APOL3; B-C: Reduced expression of APOL3 in normal mammary tissues and tumors in the GSE29431 (B) and GSE42568 (C) series; D: Reduced expression of APOL3 in normal mammary tissues and tumors from TCGA; E: Comparative analysis showing decreased APOL3 levels in tumors versus paired normal breast tissues from TCGA; F: Lower mRNA levels of APOL3 in the MCF10A cell line compared with MCF7, T47D, MDA-MB-231, MDA-MB-468, and BT549 cell lines; G: Diminished protein expression of APOL3 in the MCF10A cell line compared with MCF7, T47D, MDA-MB-231, MDA-MB-468, and BT549 cell lines; H: IHC staining for APOL3 in breast cancer and adjacent normal breast tissues. *: *p* < 0.05, **: *p* < 0.01, ***: *p* < 0.001, ****: *p* < 0.0001.

To investigate APOL3 expression levels in breast cancer, we initially consulted the GEO database and TCGA. APOL3 expression was found to be reduced in a majority of cancer types (Figure 1A). Specifically, APOL3 mRNA levels were diminished in the GSE29431 (Figure 1B) and GSE42568 (Figure 1C) series. TGCA data revealed that APOL3 expression in breast cancer tissues was lower than in normal mammary tissues (Figure 1D), with a similar downregulation observed in breast cancer tissues compared with the paired normal tissues (Figure 1E).

To further elucidate APOL3 expression in breast cancer, we employed qRT-PCR and western blotting to measure mRNA and protein levels, respectively, in various breast cancer cell lines (MCF7, T47D, MDA-MB-231, MDA-MB-468, and BT549) in comparison with the normal epithelial cell line MCF10A. Both mRNA and protein expression levels of APOL3 were found to be lower in the breast cancer cell lines than in the normal epithelial cell line (Figure 1F-G). IHC further confirmed reduced APOL3 staining intensity in breast cancer tissues versus normal mammary tissues (Figure 1H). Collectively, these data underscore the consistent finding of attenuated APOL3 expression in breast cancer.

### Low APOL3 expression in breast cancer correlates with poor prognostic outcomes

**Figure 2:**
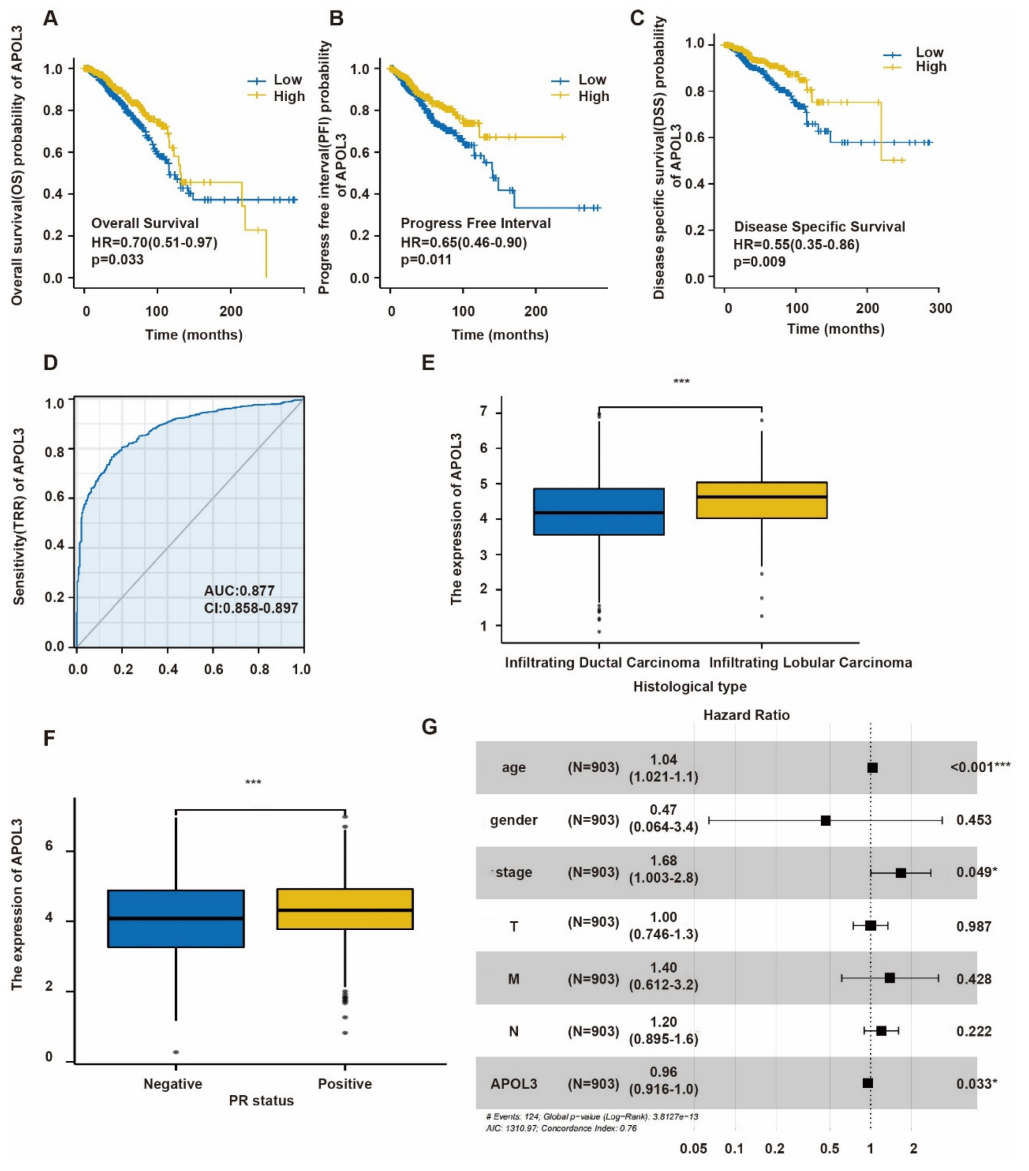
Prognostic Significance of APOL3 in Breast Cancer. A-C: Elevated APOL3 expression correlates with poor prognosis in OS (A), PFI (B), and DSS (C) of breast cancer patients; D: ROC curve for APOL3; E: Association between APOL3 expression and histological subtypes of breast cancer; F: Correlation of APOL3 expression with PR levels; G: Multivariate Cox proportional hazards regression analysis for APOL3. *: *p* < 0.05, ***: *p* < 0.001.

To elucidate the relationship between APOL3 expression and patient prognosis, Kaplan-Meier survival analysis along with the Log-Rank test was conducted using TCGA database. Low APOL3 levels were associated with a significantly reduced overall survival (OS) (*p*=0.033, HR=0.70) (Figure 2A), progress-free interval (PFI) (*p*=0.011, HR=0.65) (Figure 2B), and disease-specific survival (DSS) (*p* = 0.009, HR = 0.55) (Figure 2C). Additionally, the prognostic value of APOL3 expression was evaluated using the receiver operating characteristic (ROC) curve, showing an area under the curve (AUC) of 0.877 (Figure 2D). These findings indicate that low APOL3 expression is correlated with reduced OS, PFI and DSS.

Table 2 delineates the relationship between APOL3 expression and various clinicopathological parameters. Low APOL3 expression was more prevalent in infiltrating ductal carcinoma (IDC) than in infiltrating lobular carcinoma (ILC) (*p*<0.001) (Figure 2E) and APOL3 mRNA levels were significantly lower in the progesterone receptor (PR)-negative subgroup (*p*=0.034) (Figure 2F). Multivariate Cox regression analysis identified APOL3 expression as an independent prognostic factor in breast cancer (Figure 2G).

In summary, while APOL3 expression was not linked to TNM stage, pathologic stage, race, age, estrogen receptor (ER) and HER2 status, menopause status, or anatomic neoplasm subdivisions, it is significantly associated with histological type and PR negativity.

### APOL3 overexpression suppresses proliferation in breast cancer cells

**Figure 3:**
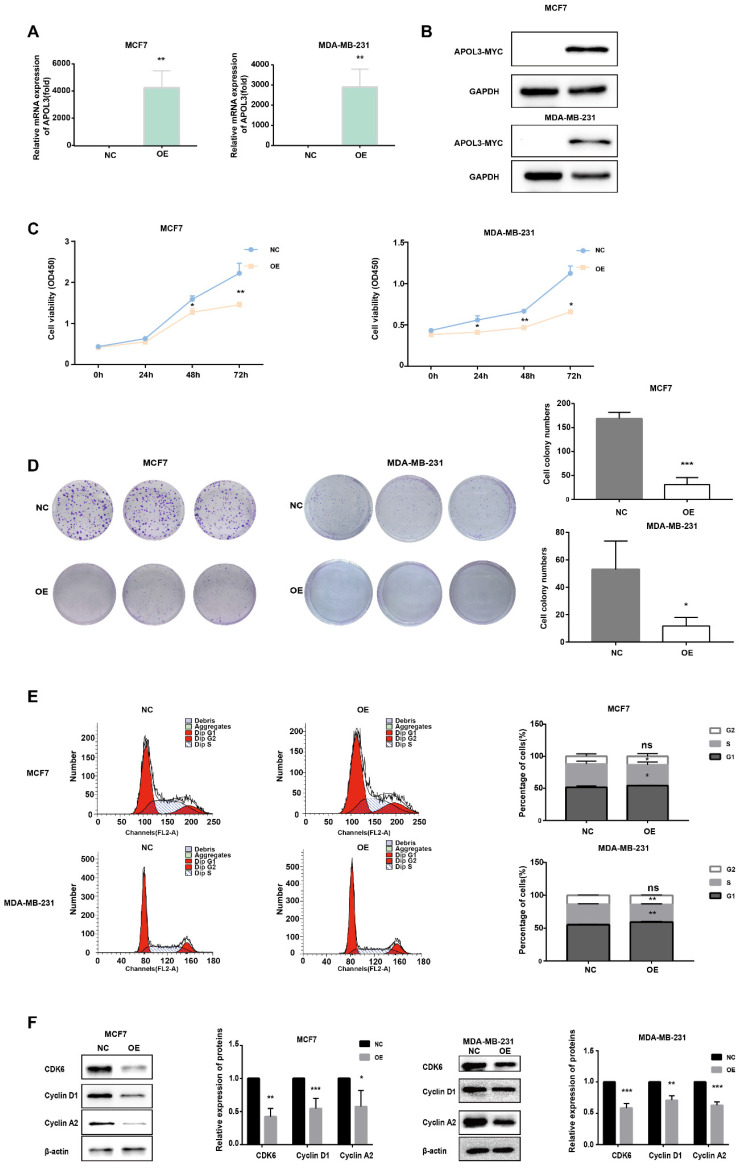
*In Vitro* Proliferative Effects of APOL3 Overexpression. A: qRT-PCR of APOL3 mRNA levels in MCF7 and MDA-MB-231 cells transfected with PEGFP-APOL3-MYC vector; B: Western blotting analysis of APOL3-MYC protein levels in MCF7 and MDA-MB-231 cells transfected with PEGFP-APOL3-MYC vector; C: CCK-8 assays monitoring cell growth rates in MCF7 and MDA-MB-231 cells transfected with PEGFP-APOL3-MYC vector; D: Evaluation and counting of colony formation; E: Flow cytometry of the cell cycle in MCF7 and MDA-MB-231 cells 24h after transfection with PEGFP-APOL3-MYC vector; F: Western blotting analysis of CDK6, Cyclin D1, and Cyclin A2 levels. *: *p* < 0.05, **: *p* < 0.01, ***: *p* < 0.001. ns: no significance.

To determine the impact of APOL3 on breast cancer cell proliferation, MCF7 cells and MDA-MB-231 cells were transfected with an APOL3 overexpression plasmid (OE-APOL3) or NC. After transfection, qRT-PCR and western blotting confirmed increased APOL3 mRNA levels (Figure 3A) and protein levels (Figure 3B).

Subsequent proliferation assays revealed that APOL3 overexpression significantly inhibited cell growth, as evidenced by the CCK-8 assay (Figure 3C). Moreover, colony formation assays demonstrated a reduction in the number of colonies containing more than 50 cells in the OE-APOL3 group compared with the NC group (Figure 3D), indicating that APOL3 restrains breast cancer cell proliferation *in vitro*.

To further explore the mechanisms underlying this proliferation inhibition, we assessed cell cycle distribution using flow cytometry. MCF7 and MDA-MB-231 cells overexpressing APOL3 exhibited an increased proportion in the G1 phase and a concomitant decrease in the S phase, with no significant difference observed in the G2 phase compared with the NC group (Figure 3E). Analysis of cell cycle related proteins by western blotting revealed that the OE-APOL3 group had significantly lower levels of CDK6, Cyclin D1, and Cyclin A2 compared with the NC group (Figure 3F).

All these findings suggest that APOL3 modulates cell cycle, thereby affecting breast cancer cell proliferation.

### APOL3 downregulation promotes breast cancer cell proliferation *in vitro*

**Figure 4:**
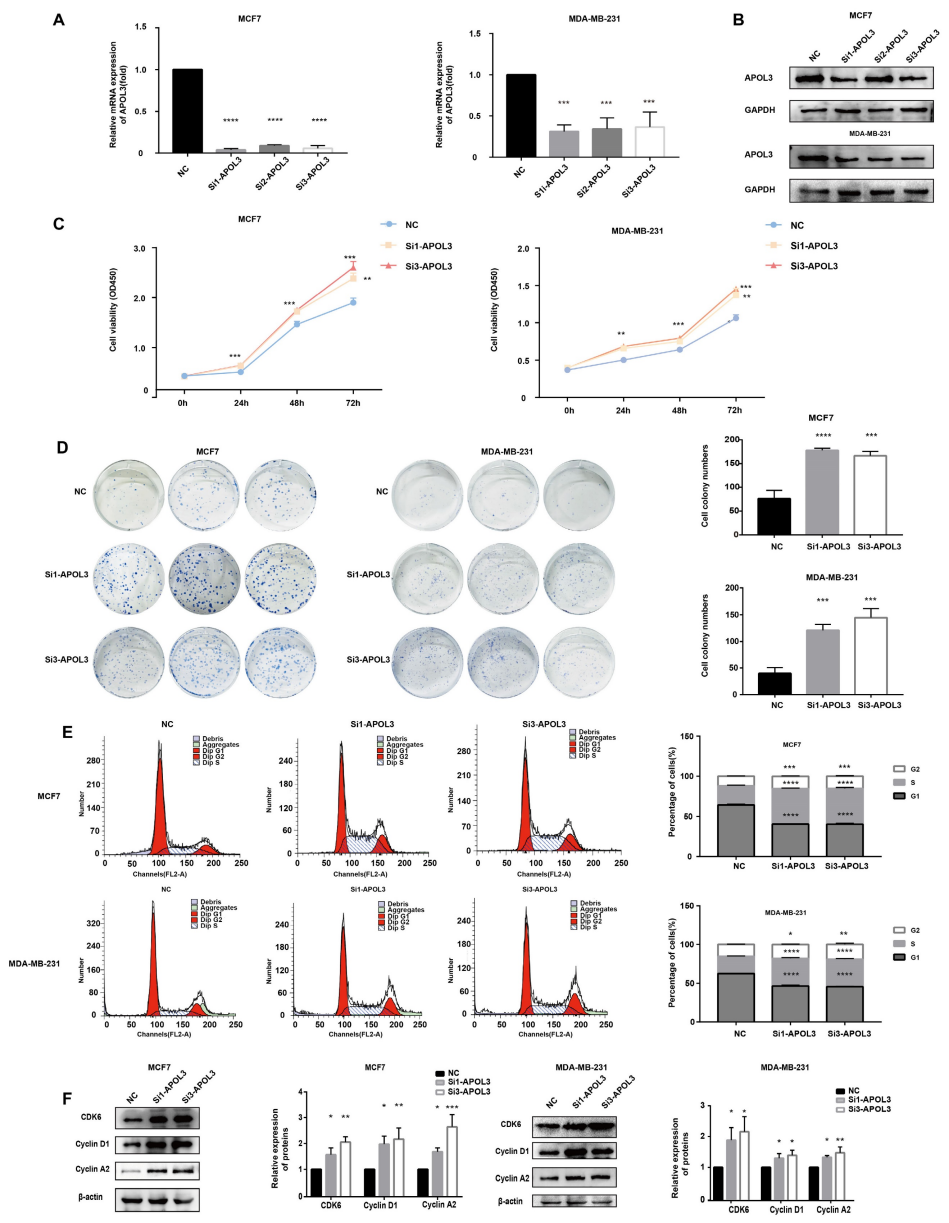
Effect of APOL3 Knockdown on *In Vitro* Proliferation. A: qRT-PCR of APOL3 mRNA levels in MCF7 and MDA-MB-231 cells transfected with siRNA; B: Western blotting analysis of APOL3 protein levels in MCF7 and MDA-MB-231 cells transfected with siRNA; C: CCK-8 assays monitoring cell growth rates in MCF7 and MDA-MB-231 cells transfected with siRNA; D: Evaluation and quantification of colony formation; E: Flow cytometry of cell cycle in MCF7 and MDA-MB-231 cells 24h after siRNA transfection; F: Western blotting analysis of CDK6, Cyclin D1, and Cyclin A2 levels. *: *p* < 0.05, **: *p* < 0.01, ***: *p* < 0.001, ****: p < 0.0001.

To assess the consequences of APOL3 downregulation on breast cancer cell proliferation, MCF7 and MDA-MB-231 cells were transfected with APOL3-targeting siRNAs (si-APOL3) and the negative control (NC). After transfection, qRT-PCR and western blotting confirmed a marked decrease in APOL3 mRNA expression (Figure 4A) and protein expression (Figure 4B) in the si-APOL3 samples. Among the three si-Apol3, si1 and si3 showed higher efficiency, were selected for further analysis.

Proliferation assays, including CCK-8 assay and colony formation, indicated that APOL3 knockdown resulted in a notable increase in cell numbers (Figure 4C) and colony counts (Figure 4D), respectively. Flow cytometry of cell cycle distribution showed a significant reduction in the G1 phase cell population and an increase in the S and G2 phase populations in the si-APOL3 group compared with the NC group (Figure 4E). Furthermore, western blotting analysis of cell cycle-related proteins showed elevated levels of CDK6, Cyclin D1, and Cyclin A2 in the Si-APOL3 group compared with the NC group (Figure 4F).

All these results suggest that the downregulation of APOL3 disrupts cell cycle regulation, thereby promoting the proliferation of breast cancer cells *in vitro*.

### APOL3 overexpression suppresses breast cancer tumorigenesis *in vivo*

**Figure 5:**
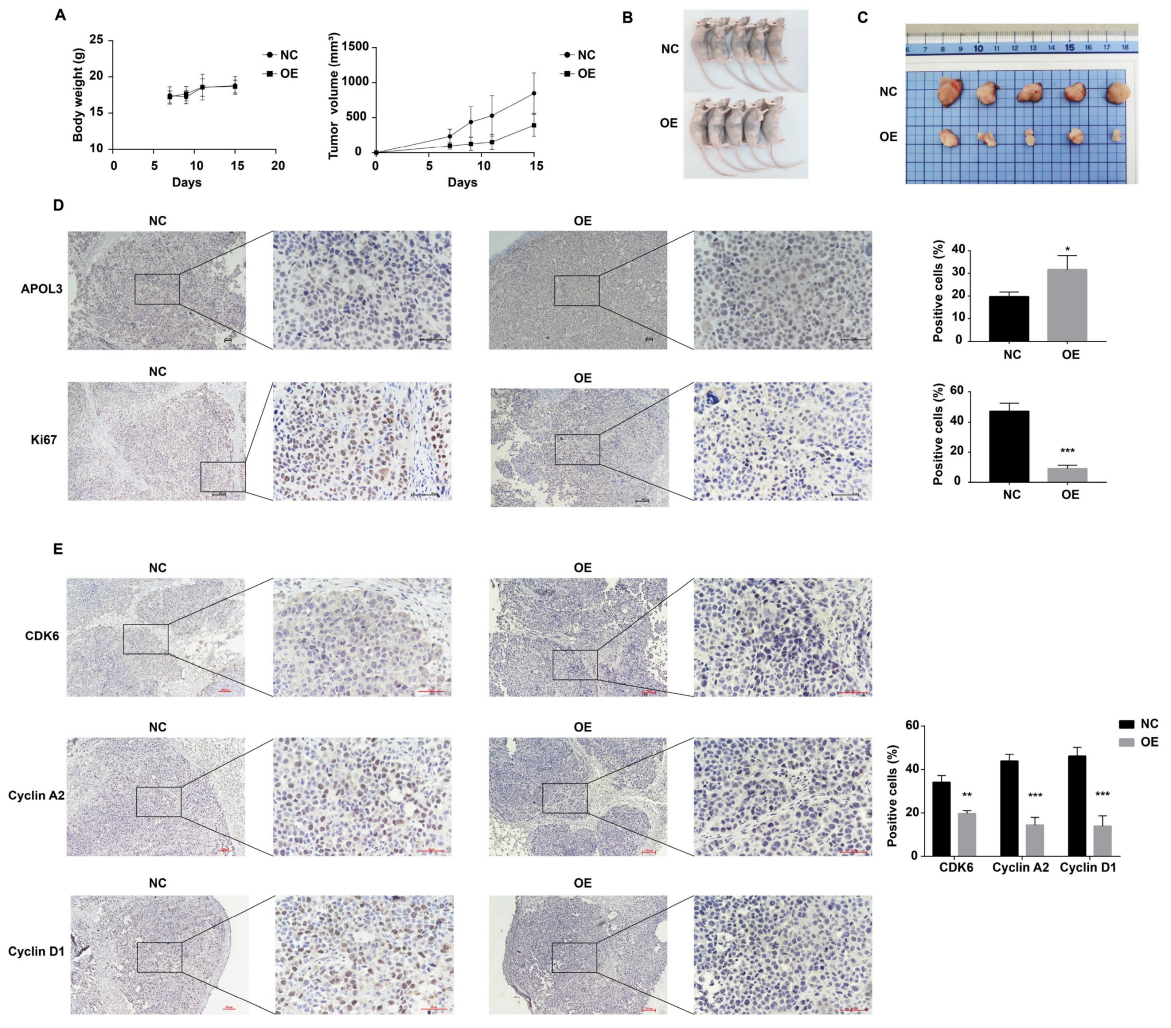
APOL3 Overexpression and Its effects on Tumor Growth *In Vivo*. A: Growth curves of tumors in nude mice inoculated with APOL3-overexpressing MCF7 cells; B: Representative images of nude mice after tumor formation; C: Macroscopic view of excised tumors; D: IHC staining of tumor tissue sections. E: IHC staining for cell cycle related proteins in tumor tissue sections. *: *p* < 0.05, **: *p* < 0.01, ***: *p* < 0.001, ****: *p* < 0.0001.

To evaluate the inhibitory effects of APOL3 overexpression on breast cancer tumorigenesis *in vivo*, MCF7 cells transfected with OE-APOL3 and NC, followed by G418 selection, were subcutaneously inoculated into nude mice. Tumor volumes were measured every two days. The resulting tumor growth curves are shown in Figure 5A.

Notably, the mice in the OE-APOL3 group developed tumors that were significantly smaller in size compared with the NC group, with both tumor volume and weight being significantly reduced in the OE-APOL3 group (Figures 5B and 5C). IHC staining for Ki-67 revealed a pronounced reduction in tumor cell proliferation in the OE-APOL3 group with statistically significant (Figure 5D), supporting the role of APOL3 overexpression as a deterrent to breast cancer cell tumorigenesis in a live model. IHC assay was also used to detect the expression of CDK6, Cyclin D1 and Cyclin A2 in NC and OE-APOL3 group, which showed a decreased level in OE-APOL3 group (Figure 5E). In conclusion, overexpression of APOL3 inhibits the growth of breast cancer *in vivo*.

### APOL3 promotes p53 signaling through interaction with Y-box-binding protein 1 (YBX1)

**Figure 6:**
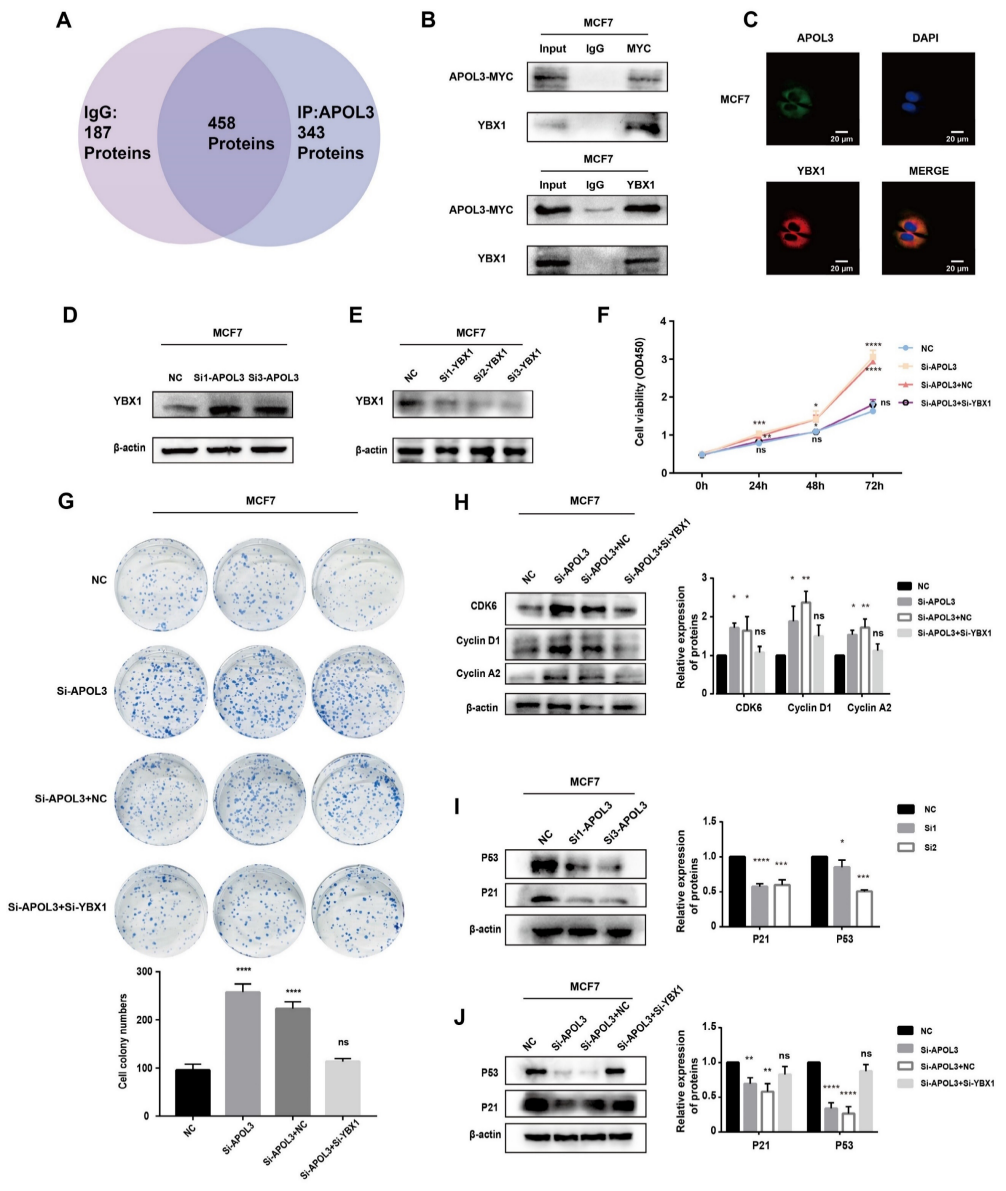
APOL3 Exerts Significant Effects via the P53 pathway. A: Mass spectrometry results; B: CO-IP confirming APOL3-YBX1 interaction; C: Immunofluorescence assay of APOL3 and YBX1. D: Western blotting analysis of YBX1 alterations following APOL3 knockdown; E: Verification of YBX1 knockdown efficiency by western blotting; F-G: Reversal of si-APOL3-induced cell proliferation by YBX1 knockdown, assessed by CCK8 assay (F) and colony formation counting (G); H: Western blotting analysis of CDK6, Cyclin D1, and Cyclin A2 levels; I: APOL3 knockdown diminishes P53 and P21 expression; J: Restoration of P53 and P21 levels upon concurrent knockdown of YBX1 and APOL3. *: *p* < 0.05, **: *p* < 0.01, ***: p < 0.001, ****: *p* < 0.0001. ns: no significance.

To elucidate the potential mechanisms by which APOL3 influences breast carcinogenesis, mass spectrometry analysis was conducted on MCF7 cells which overexpressed APOL3. This analysis aimed to identify differentially associated proteins between IgG and APOL3-IP groups. YBX1 was found to be highly associated with APOL3, being present in the APOL3-IP group but absent in the IgG group (Figure 6A). The interaction between YBX1 and APOL3 was further validated by CO-IP, confirming the binding of APOL3 to YBX1 (Figure 6B). Immunofluorescence assay was used to confirm the co-localization of APOL3 and YBX1 in MCF7 (Figure 6C).

Subsequent western blotting revealed that YBX1 expression increased in the si-APOL3 MCF7 cells (Figure 6D). To investigate whether APOL3 exerts its biological functions through YBX1, a rescue assay was performed in MCF7 cells. YBX1 expression was knocked down using specific siRNAs (si-YBX1), and the efficiency of this knockdown was confirmed by western blotting (Figure 6E). CCK8 and colony formation assays showed that YBX1 knockdown significantly counteracted the proliferation increase induced by APOL3 knockdown in breast cancer cells (Figures 6F-G). Furthermore, western blotting showed that the downregulation of YBX1 mitigated the increase in CDK6, CyclinD1, and Cyclin A2 protein expression induced by si-APOL3 (Figure 6H).

**Figure 7:**
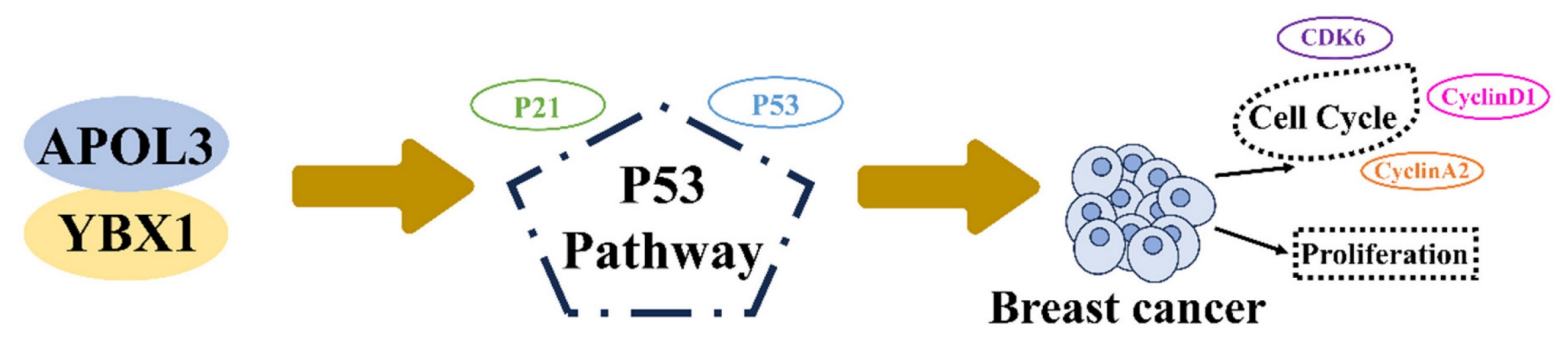
APOL3 modulates cell cycle and proliferation of breast cancer through binding to YBX1 through P53 pathway.

Previous studies have implicated the role of YBX1 in the regulation of the p53 pathway 18-20. To examine alterations in P53 signaling, western blotting was used to measure the expression of P53 and its downstream effector, P21. Knockdown of APOL3 was found to reduce the expression of both P53 and P21 in MCF7 and MDA-MB-231 cells (Figure 6I). However, simultaneous knockdown of YBX1 and APOL3 restored the expression levels of P53 and P21 in MCF7 cells (Figure 6J), indicating that YBX1 knockdown can reverse the pro-carcinogenic effects of APOL3 knockdown. These findings indicate that APOL3 may inhibit breast cancer progression by enhancing P53 signaling pathway through its interaction with YBX1 (Figure 7).

## Discussion

Apolipoproteins (APOs) have been studied extensively in cardiovascular diseases, however, their roles in cancer remain underexplored 21-23. APOs have the capability to bind lipids, facilitating their transport through the body for metabolic process and utilization. In humans, APOs are categorized into 10 subgroups, from APOA to APOJ 14. The APOL family, comprising APOL1 through APOL6, shares structural and functional similarities with the Bcl-2 family 8. Chidiac M et al. highlighted an elevated expression of APOL1 in papillary thyroid carcinoma 11. Chu J et al. developed a risk score staging system, indicating that a higher level of APOL2 correlate with poorer bladder cancer prognosis 12. The presence of the BH3 domain within APOL6, a critical component in Bcl-2 family proteins, has also been reported 24. Our study revealed that APOL3 expression is diminished in breast cancer tissues compared with normal mammary tissues. To quantify expression levels, we employed qRT-PCR, western blotting and immunohistochemistry assay.

Cancer is characterized by dysregulated cell proliferation due to the disruption of pathways that govern this process. Additionally, cell cycle dysregulation is a contributing factor to tumorigenesis 25. Cell cycle, a widely conserved and precise process, includes four phases: G0/G1 (gap 1), S (DNA synthesis), G2 (gap 2), and M (mitosis), along with multiple checkpoints. Normally, the cells repair damaged DNA during the G1/S phase, but cancer cells often exhibit a compromised G1/S checkpoint and rely on the G2/M phase for DNA repair 26, 27. In our study, APOL3 overexpression induced cell cycle alterations, characterized by an extended G1 phase, a shortened S phase, and reduced levels of cell cycle related proteins. Conversely, APOL3 knockdown resulted in a shortened G1 phase and elongated S and G2 phases. Overexpression of APOL3 inhibited breast cancer cell proliferation, while its knockdown facilitates tumorigenesis.

YBX1, a 36 kDa multifunctional protein, is comprised of an alanine/prolyl-rich N-terminal domain (A/P domain), a widely conserved cold shock domain (CSD), and a sizable C-terminal domain (CTD). As a DNA/RNA binding protein with nucleic acid chaperone properties, YBX1 interacts with various proteins 28 and is pivotal in transcription, translation, RNA splicing, and DNA repair 29. Recent studies have underscored YBX1's significant role in tumor development. It is frequently overexpressed in cancers, driving tumorigenesis by stimulating cell proliferation, inhibiting apoptosis, promoting epithelial-mesenchymal transition (EMT), enhancing cell invasiveness, and increasing treatment resistance 30, 31. Due to its important role in tumors, YBX1 is considered a key marker of malignancy and a promising molecular target for cancer therapy 32. The in-depth exploration of YBX1 opens avenues for novel therapeutic strategies, offering new hope in cancer treatment.

Our study demonstrates that APOL3 interacts with YBX1 and modulates the expression of YBX1 by mass spectrum, CO-IP, immunofluorescence assay and western blotting. Furthermore, the effects of APOL3 knockdown on breast cancer cells were reversible through concurrent knockdown of YBX1. Prior investigations into the P53 pathway have revealed that YBX1 can influence P53 expression 19, 31, 33, 34. Through the assessment of P53 and P21 protein levels within this pathway, we discovered that APOL3 knockdown diminished their expression, whereas concurrent YBX1 knockdown restored it. This indicates that APOL3's impact on breast cancer may be mediated by the P53 pathway through modulating the expression of YBX1. Furthermore, we are delving into the precise molecular interactions involved, which requires additional experiments in the future.

Our study is not without limitations: The exact mechanisms by which APOL3 and YBX1 interact and the means by which APOL3 regulates YBX1 remain to be elucidated in future research.

In conclusion, our findings highlight that APOL3 expression is reduced in breast cancer cells and APOL3 acts as a tumor suppressor gene, inhibiting breast cancer proliferation through regulation of cell cycle via the P53 pathway. Our results position APOL3 as a potential prognostic biomarker for breast cancer.

## Figures and Tables

**Table 1:** Gene primer sequences

Gene	Forward primer	Reverse primer
APOL3	CACCATTTACAGCAGGGACGAGTC	ATGTGTATGAGTGCTCCACGATGC
β-actin	CTGGCCGGGACCTGACT	TCCTTAATGTCACGCACGATTT

**Table 2:** Relationship between APOL3 expression and clinicopathologic parameters of breast cancer patients

Parameter	Category	Low expression of APOL3	High expression of APOL3	*p*
n		541	542	
T stage, n (%)	T1	136 (12.6%)	141 (13.1%)	0.392
	T2	316 (29.3%)	313 (29%)	
	T3	65 (6%)	74 (6.9%)	
	T4	22 (2%)	13 (1.2%)	
N stage, n (%)	N0	259 (24.3%)	255 (24%)	0.905
	N1	178 (16.7%)	180 (16.9%)	
	N2	54 (5.1%)	62 (5.8%)	
	N3	38 (3.6%)	38 (3.6%)	
M stage, n (%)	M0	450 (48.8%)	452 (49%)	0.516
	M1	8 (0.9%)	12 (1.3%)	
Pathologic stage, n (%)	Stage I	89 (8.4%)	92 (8.7%)	0.820
	Stage II	309 (29.2%)	310 (29.2%)	
	Stage III	122 (11.5%)	120 (11.3%)	
	Stage IV	7 (0.7%)	11 (1%)	
Race, n (%)	Asian	27 (2.7%)	33 (3.3%)	0.647
	Black or African American	94 (9.5%)	87 (8.8%)	
	White	380 (38.2%)	373 (37.5%)	
Age, n (%)	<=60	300 (27.7%)	301 (27.8%)	1.000
	>60	241 (22.3%)	241 (22.3%)	
Histological type, n (%)	Infiltrating Ductal Carcinoma	408 (41.8%)	364 (37.3%)	< 0.001
	Infiltrating Lobular Carcinoma	76 (7.8%)	129 (13.2%)	
ER status, n (%)	Negative	133 (12.9%)	107 (10.3%)	0.089
	Indeterminate	1 (0.1%)	1 (0.1%)	
	Positive	383 (37%)	410 (39.6%)	
PR status, n (%)	Negative	189 (18.3%)	153 (14.8%)	0.034
	Indeterminate	2 (0.2%)	2 (0.2%)	
	Positive	325 (31.4%)	363 (35.1%)	
HER2 status, n (%)	Negative	264 (36.3%)	294 (40.4%)	0.852
	Indeterminate	5 (0.7%)	7 (1%)	
	Positive	77 (10.6%)	80 (11%)	
Menopause status, n (%)	Pre	109 (11.2%)	120 (12.3%)	0.478
	Peri	17 (1.7%)	23 (2.4%)	
	Post	356 (36.6%)	347 (35.7%)	
Anatomic neoplasm subdivisions, n (%)	Left	281 (25.9%)	282 (26%)	1.000
	Right	260 (24%)	260 (24%)	
